# Design of co-crystals/salts of some Nitrogenous bases and some derivatives of thiophene carboxylic acids through a combination of hydrogen and halogen bonds

**DOI:** 10.1186/1752-153X-8-20

**Published:** 2014-03-22

**Authors:** Samson Jegan Jennifer, Packianathan Thomas Muthiah

**Affiliations:** 1 School of Chemistry, Tiruchirappalli 620024 Tamil Nadu, India

**Keywords:** 5-chlorothiophen-2-carboxylic acid, Halogen bonding, Bipyridine, Pyrimidine, Salts, Cocrystal

## Abstract

**Background:**

The utility of N-heterocyclic bases to obtain molecular complexes with carboxylic acids is well studied. Depending on the solid state interaction between the N-heterocyclic base and a carboxylic acid a variety of neutral or ionic synthons are observed. Meanwhile, pyridines and pyrimidines have been frequently chosen in the area of crystal engineering for their multipurpose functionality. HT (hetero trimers) and LHT (linear heterotetramers) are the well known synthons that are formed in the presence of pyrimidines and carboxylic acids.

**Results:**

Fourteen crystals involving various substituted thiophene carboxylic acid derivatives and nitrogenous bases were prepared and characterized by using single crystal X-ray diffraction. The 14 crystals can further be divided into two groups [**1a-7a**], [**8b-14b**] based on the nature of the nitrogenous base. Carboxylic acid to pyridine proton transfer has occurred in 3 compounds of each group. In addition to the commonly occurring hydrogen bond based pyridine/carboxylic acid and pyrimidine/carboxylic acid synthons which is the reason for assembly of primary motifs, various other interactions like Cl…Cl, Cl…O, C–H…Cl, C-H…S add additional support in organizing these supermolecules into extended architectures. It is also interesting to note that in all the compounds π-π stacking occurs between the pyrimidine-pyrimidine or pyridine-pyridine or acid-acid moieties rather than acid-pyrimidine/pyridine.

**Conclusions:**

In all the compounds (**1a-14b**) either neutral O–H…N_pyridyl/pyrimidine_ or charge-assisted N_pyridinium_-H…O_carboxylate_ hydrogen bonds are present. The HT (hetero trimers) and LHT (linear heterotetramers) are dominant in the crystal structures of the adducts containing N-heterocyclic bases with two proton acceptors (**1a-7a**). Similar type supramolecular ladders are observed in 5TPC44BIPY (**8b**), TPC44BIPY (**9b**), TPC44TMBP (**11b**). Among the seven compounds [**8b**-**14b**] the extended ligands are linear in all except for the TMBP (**10b, 11b, 12b**). The structure of each compound depends on the dihedral angle between the carboxyl group and the nitrogenous base. All these compounds indicate three main synthons that regularly occur, namely linear heterodimer (HD), heterotrimer (HT) and heterotetramer (LHT).

## Background

The utilization of intermolecular interactions for directed self assembly in order to understand their strength, directionality as well as distance is perhaps the main goal of supramolecular chemistry and crystal engineering [1-6]. The identification of various commonly occurring synthons between two functional groups not only adds to knowledge but also simplifies the design and prediction of such supramolecular assemblies [7-9]. These interactions involve electrostatic, hydrogen bonding, Van der Waals and pi-pi stacking interactions. The perfect example that emphasizes the importance of hydrogen bonding is provided by Mother Nature in the form of complementary base pairing of the double helix of DNA [10,11]. Recently halogen bonding is a paradigm that complements the role of hydrogen bonding and its importance in formation of crystal structures has also been emphasized [12-14]. Also in recent years, the advances in this field have enabled the design and synthesis of molecules of pharmaceutical importance with improved physico-chemical properties [15-18]. The utility of N-heterocyclic bases to obtain molecular complexes with carboxylic acids is well studied [19-23]. Depending on the solid state interaction between the N-heterocyclic base and a carboxylic acid a variety of neural or ionic synthons are observed.

**Scheme 1:**
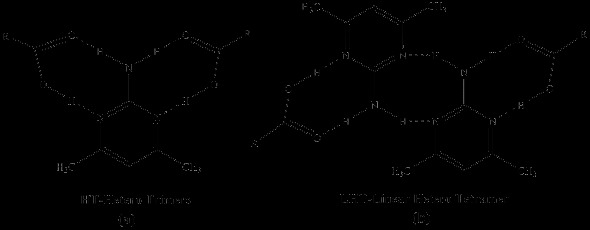
Supramolecular heterosynthons that can be formed between carboxylic acids heterocyclic nitrogen of the AMPY: (a) hetero trimer (HT) (Synthon type-I) (b) linear hetero tetramer (LHT) (Synthon type-II).

**Scheme 2:**
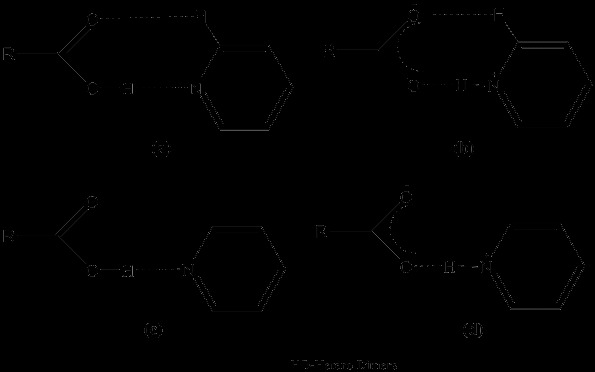
Supramolecular hetero dimers that can be formed between carboxylic acids/carboxylate and heterocyclic nitrogen: (a) carboxylic acid-aromatic nitrogen with R_2_^2^(7) synthon (Synthon type-III)(b) pyridinium-carboxylate with R_2_^2^(7) synthon (Synthon type-IV)(c) carboxylic acid-aromatic nitrogen with single point heterosynthon (Synthon type-V)and (d) pyridinium-carboxylate with single point heterosynthon (Synthon type-VI) **Supramolecular hetero dimers that can be formed between carboxylic acids/carboxylate and heterocyclic nitrogen: (a) carboxylic acid-aromatic nitrogen with R**_2_^**2**^**(7) synthon (Synthon type-III)(b) pyridinium-carboxylate with R**_**2**_^**2**^**(7) synthon (Synthon type-IV)(c) carboxylic acid-aromatic nitrogen with single point heterosynthon (Synthon type-V)and (d) pyridinium-carboxylate with single point heterosynthon (Synthon type-VI).**

Meanwhile, pyridines and pyrimidines have been frequently chosen in the area of crystal engineering for their multipurpose functionality. HT (hetero trimers) and LHT (linear heterotetramers) are the well known synthons that are formed in the presence of pyrimidines and carboxylic acids (Scheme 1). Especially the bipyridyl species with the robust hydrogen bonding sites and high molecular symmetry make it a versatile building block in building crystalline materials [23-26]. For the sake of further investigation, it is worthwhile to analyze and compare complex structures based on these pyridines and pyrimidines with rigid building blocks such as TPC, 5-TPC and TDC (TPC = Thiophene 2-carboxylic acid, 5-TPC = 5-Chloro thiophene 2- carboxylic acid, TDC-Thiophene dicarboxylic acid). 5-TPC has been an interesting ligand to us due to its hydrogen bonding ability as well as formation of interesting Cl…Cl and C-H…Cl halogen bonding interactions [23,27-30]. Beyond this, the aim of this work is to compare the differences that occur during the interchange of pyridines/pyrimidines and to list out the commonly occurring motifs. In our current investigation, the expected carboxyl-pyridyl heterosynthon [O–H…N_pyridyl_] with graph set notation R_2_^2^(7) is completely absent (Scheme 2). Instead of the R_2_^2^(7) heterosynthon a single point synthon is observed (Scheme 2). This R_2_^2^(7) hetero synthon is the most commonly occuring and the reliable recognition pattern between the carboxyl and pyridyl groups [6,31,32].

## Results and discussion

**Figure 1:**
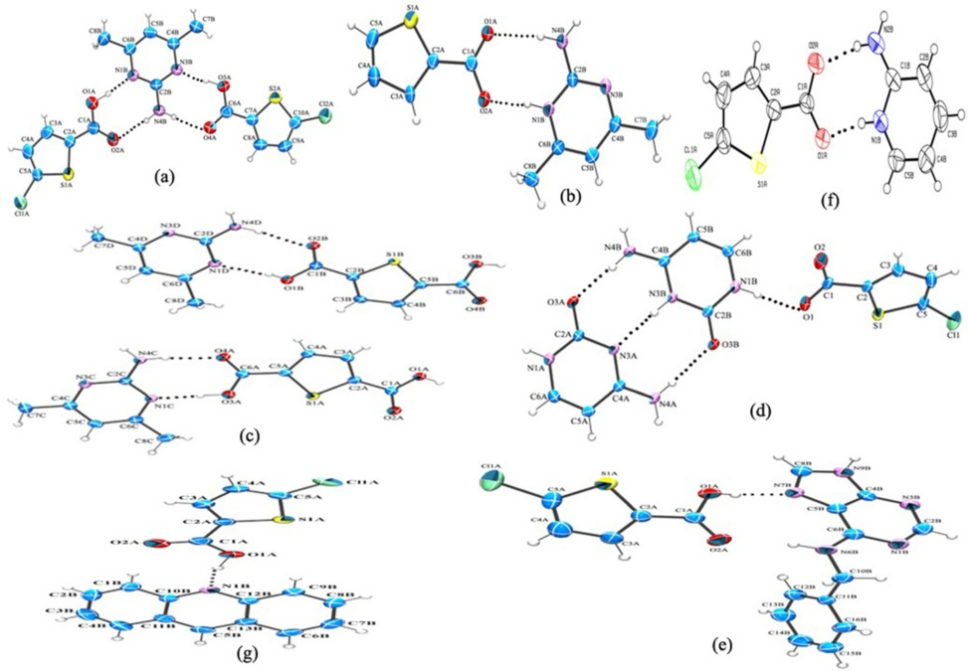
**(a-g) ORTEP views of compounds 1a-7a showing the atom-numbering scheme.** Displacement ellipsoids drawn at 50% probability level for all non hydrogen atoms and H atoms are shown as small spheres of arbitrary radii.

**Figure 2:**
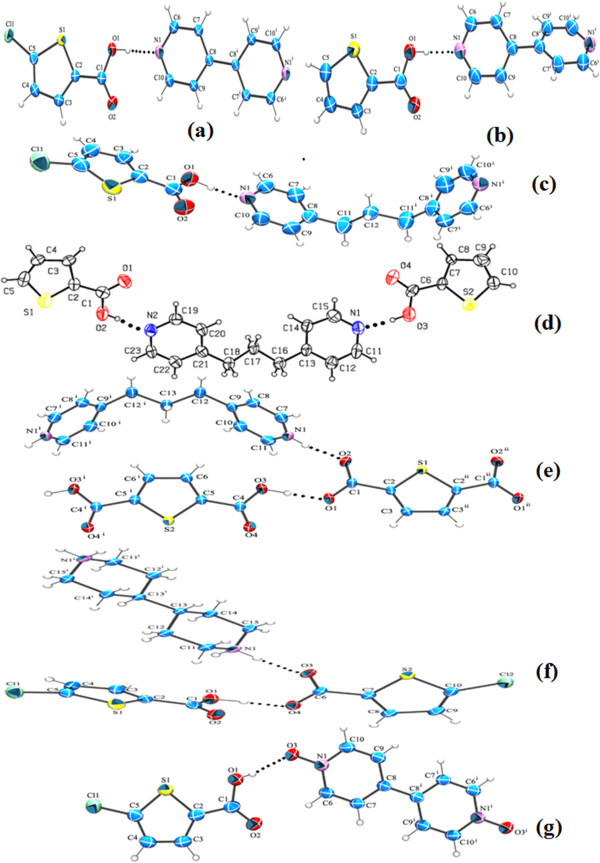
**(a-g) ORTEP views of compounds 8b-14b showing the atom-numbering scheme.** Displacement ellipsoids drawn at 50% probability level for all non hydrogen atoms and H atoms are shown as small spheres of arbitrary radii.

**Scheme 3:**
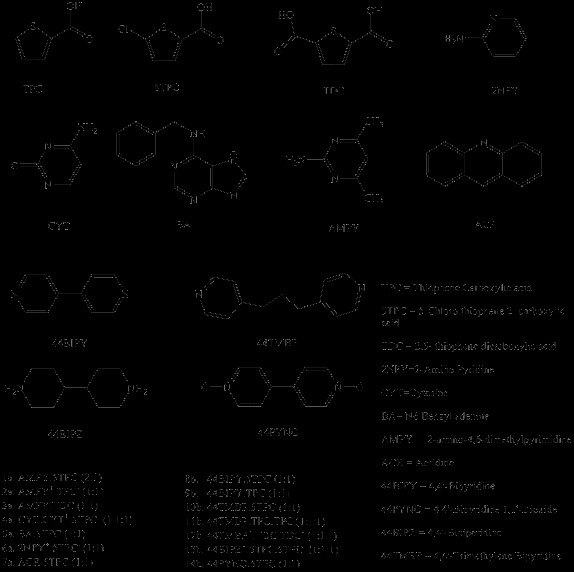
Molecular structures of components used in 1–14 Molecular structures of components used in 1–14.

Crystallographic data for the compounds (**1a-7a**) and (**8b-14b**) are summarized in (Tables 1 and 2) respectively. The hydrogen bonding parameters for the compounds (**1a-7a**) and (**8b-14b**) are listed in (Tables 3 and 4) respectively. ORTEP views of compounds (**1a-7a**) and (**8b-14b**) are shown in (Figures 1 and 2) respectively. Detailed structural description of all the co-crystals and salts are given below in succession. The details of the co-formers are given in (Scheme 3).

**Table 1:** Crystallographic data for structures 1a - 7a

**Sample code**	**5TPCAMPY (1a)**	**TPCAMPY (2a)**	**TDCAMPY (3a)**	**5TPCCYT (4a)**	**5TPCBA (5a)**	**5TPC2NPY (6a)**	**5TPCACR (7a)**
Empirical Formula	C_6_ H_9_ N_3_, 2(C_5_ H_3_ Cl S)	C_6_ H_10_ N_3_, C_5_ H_3_ O_2_ S	C_6_ H_9_ N_3_, C_6_ H_4_ O_4_ S	C_4_ H_6_ N_3_ O, C_4_ H_5_ N_3_ O, C_5_ H_2_ Cl O_2_ S	C_12_ H_11_ N_5_, C_5_ H_3_ Cl O_2_ S	C_5_ H_7_ N_2_, C_5_ H_2_ Cl O_2_ S	C_13_ H_9_ N, C_5_ H_3_ Cl O_2_ S
Formula weight	448.35	251.31	295.32	384.81	387.85	256.71	341.81
Temp, K	296	296	296	296	296	296	296
λ (Å)	0.71073	0.71073	0.71073	0.71073	0.71073	0.71073	0.71073
Crystal system	Monoclinic	Monoclinic	Monoclinic	Triclinic	Triclinic	Monoclinic	Triclinic
Space group	P2_1_/c	P2_1_/c	P2_1_/c	P-1	P-1	P2_1_/c	P-1
a (Å)	7.9724(3)	6.5830(2)	7.6474(3)	7.2399(1)	4.9941(3)	8.4777(8)	7.6417(1
b (Å)	11.4924(3)	25.0842(7)	23.8704(7)	7.4140(1)	11.3127(7)	12.4062(12)	8.8984(2)
c (Å)	21.6804(7)	9.6092(3)	14.7634(5)	17.4125(3)	16.3754(9)	11.9771(10)	12.1882(2)
α(º)	90	90	90	89.751(1)	106.595(4)	90	73.569(1)
β (º)	96.742(2)	128.170(2)	92.372(2)	88.462(1)	92.393(4)	111.541(6)	87.200(1)
γ (º)	90	90	90	61.449(1)	100.126(4)	90	86.598(1)
V (Å^3^)	1972.67(11)	1247.48(7)	2692.70(16)	820.65(2)	868.66(9)	1171.72(19)	793.09(2)
Z	4	4	8	2	2	4	2
Final R_1_ index [I > 2σ(I)]	0.0390	0.0757	0.0451	0.0444	0.0585	0.0398	0.0401
wR_2_ (all data)	0.0891	0.2970	0.1570	0.1331	0.1345	0.1242	0.1070
Largest difference in peak and hole (e Å^-3^)	-0.19, 0.16	-0.57, 0.90	-0.23, 0.27	-0.54, 0.70	-0.23, 0.46	-0.49, 0.82	-0.21, 0.18

**Table 2:** Crystallographic data for Structures 8b-14b

**Sample code**	**5TPC44BIPY (8b)**	**TPC44BIPY (9b)**	**5TPC44TMBP (10b)**	**TPC44TMBP (11b)**	**TDC44TMBP (12b)**	**5TPC44BIPZ (13b)**	**5TPC44PYNO (14b)**
Empirical Formula	C_10_ H_8_ N_2_, 2(C_5_ H_3_ Cl O_2_ S)	C_10_ H_8_ N_2_, 2(C_5_ H_4_ O_2_ S)	C_13_ H_14_ N_2_, 2(C_5_ H_3_ Cl O_2_ S)	C_13_ H_15_ N_2_, C_5_ H_4_ O_2_ S, C_5_ H_3_ O_2_ S	C_13_ H_16_ N_2_, C_6_ H_4_ O_4_ S, C_6_ H_2_ O_4_ S	C_10_ H_22_ N_2_, 2(C_5_ H_3_ Cl O_2_ S), 2(C_5_ H_2_ Cl O_2_ S)	C_10_ H_8_ N_2_ O_2_, 2(C_5_ H_3_ Cl O_2_ S)
Formula weight	481.37	412.49	523.45	454.57	542.59	818.66	513.37
Temp, K	296	296	296	296	296	296	296
λ (Å)	0.71073	0.71073	0.71073	0.71073	0.71073	0.71073	0.71073
Crystal system	Monoclinic	Orthorhombic	Monoclinic	Orthorhombic	Monoclinic	Triclinic	Monoclinic
Space group	P2_1_/c	Pbcn	C2	Pna2_1_	P2_1_/m	P-1	P2_1_/c
a (Å)	3.8843(1)	26.1893(5)	24.9390(4)	16.8789(4)	6.9798(1)	8.3133(1)	6.8923(5)
b (Å)	28.0949(5)	7.4673(2)	4.7213(1)	5.9661(1)	18.8881(4)	9.5054(1)	13.0669(8)
c (Å)	9.6835(2)	10.0668(2)	10.4626(2)	22.7337(5)	9.8801(2)	11.9923(2)	11.9438(7)
α(º)	90	90	90	90	90	90.270(1)	90
β (º)	105.086(1)	90	93.980(2)	90	110.087(1)	105.535(1)	95.316(5)
γ (º)	90	90	90	90	90	97.778(1)	90
V (Å^3^)	1020.33(4)	1968.70(8)	1228.94(4)	2289.31(8)	1223.31(4)	903.79(2)	1071.04(12)
Z	2	4	2	4	2	1	2
Final R1 index [I > 2σ(I)]	0.0385	0.0400	0.0371	0.0608	0.0335	0.0400	0.0452
wR_2_ (all data)	0.1040	0.1258	0.1061	0.1904	0.0909	0.1519	0.1336
Largest difference in peak and hole (e Å^-3^)	-0.18, 0.33	-0.27, 0.17	-0.21, 0.22	-0.42, 0.66	-0.22, 0.20	-0.27, 0.34	-0.33, 0.27

**Table 3:** Hydrogen bond metrics for compounds 1a-7b

**D---H....A**	**H…A (Ǻ)**	**D…A (Ǻ)**	**D -H…A**	**Symmetry operation**
**5TPC AMPY (1a)**				
O1A-H1A∙∙∙N1B	1.66(4)	2.636(4)	172(3)	
O3A-H3A∙∙∙N3B	1.70(4)	2.682(3)	178(4)	
N4B-H3C∙∙∙O2A	2.08	2.928(4)	170	
N4B-H3D∙∙∙O4A	2.05	2.902(4)	171	
C8A-H8A∙∙∙O4A	2.55	3.419(5)	155	1-x,-y,1-z
C8B-H8E∙∙∙O4A	2.60	3.495(5)	156	1-x,1-y,1-z
**C-X…Cg**	**X…Cg (Å)**	**C-X..Cg (°)**	**Y..Cg (Å)**	**Symmetry code**
C5A-Cl1A Cg3	3.7555(19)	92.33(12)	4.190(4)	-1 + X,1/2-Y,-1/2 + Z
C10A-Cl2A Cg2	3.9862(19)	88.75(14)	4.302(4)	1 + X,1/2-Y,1/2 + Z
**TPCAMPY(2a)**				
N1B-H1B∙∙∙O2A	1.74	2.593(4)	171	
N4B-H4C∙∙∙N3B	2.19	3.043(5)	171	-x,1-y,2-z
N4B-H4D∙∙∙O1A	2.08	2.912(4)	164	
C5A-H5A∙∙∙O1A	2.51	3.417(4)	165	x,1/2-y,-1/2 + z
**TDCAMPY(3a)**				
N4C-H4E∙∙∙O2A	2.07	2.926(3)	173	1-x,-1/2 + y,3/2-z
N4C-H4F∙∙∙O4A	2.00	2.853(3)	171	
N4D-H4H∙∙∙O2B	2.04	2.897(3)	174	
N4D-H4I∙∙∙O4B	2.08	2.933(3)	171	2-x,-1/2 + y,1/2-z
O1A-H11A∙∙∙N3C	1.83(3)	2.608(3)	166(3)	1-x,1/2 + y,3/2-z
O1B-H11B∙∙∙N1D	1.96(3)	2.660(3)	171(3)	
O3A-H33A∙∙∙N1C	1.67(3)	2.643(3)	177(3)	
O3B-H33B∙∙∙N3D	1.69(3)	2.636(3)	167(3)	2-x,1/2 + y,1/2-z
**5TPC CYT(4a)**				
N1A-H1A∙∙∙O2	1.96	2.759(3)	154	1-x,2-y,1-z
N1B-H1B∙∙∙O1	1.76	2.6194(18)	172	
N3B-H3B∙∙∙N3A	2.00	2.8586(17)	177	
N4A-H4C∙∙∙O3B	2.06	2.9234(19)	176	
N4A-H4D∙∙∙O3A	2.15	2.8632(19)	140	x,-1 + y,z
N4B-H4E∙∙∙O3A	1.92	2.7836(19)	177	
N4B-H4F∙∙∙O3B	2.09	2.8626(18)	149	x,1 + y,z
**C-X…Cg**	**X…Cg (Å)**	**C-X..Cg (°)**	**Y..Cg (Å)**	**Symmetry code**
C5-Cl1 Cg3	3.6501(10)	97.03(7)	4.220(2)	-X,1-Y,-Z
C1-O1 Cg2	3.4019(16)	141.27(12	4.460(2)	-X,2-Y,1-Z
C2A-O3A Cg1	3.4771(14)	69.46(9)	3.2563(16)	1-X,2-Y,1-Z
**5TPC BA(5a)**				
N1 –H1∙∙∙O1	2.00(6)	2.886(7)	169(7)	-1 + x,y,z
O2 -H2A∙∙∙N5	1.85(6)	2.631(6)	173(8)	1 + x,y,z
N4 –H4 ∙∙∙N3	1.89(6)	2.886(7)	169(6)	1-x,-y,1-z
C8 –H8 ∙∙∙N1	2.54	2.875(8)	101	
C11-H11∙∙∙O2	2.60	3.479(9)	158	x,1 + y,z
**C-X…Cg**	**X…Cg (Å)**	**C-X..Cg (°)**	**Y..Cg (Å)**	**Symmetry code**
C5A-Cl1A Cg3	3.874(3)	127.9(3)	5.105(7)	-1-X,1-Y,2-Z
C1A-O2A Cg1	3.945(5)	63.4(3)	3.571(6)	-1 + X,Y,Z
C1A-O2A Cg5	3.962(5)	69.1(3)	3.707(6)	1 + X,Y,Z
**5TPC2NPY(6a)**				
N1B-H1B∙∙∙O1A	1.82	2.6667(17)	169	
N2B-H2C∙∙∙O2A	1.94	2.7861(19)	169	
N2B-H2D∙∙∙O1A	2.03	2.8873(19)	172	x,3/2-y,1/2 + z
C2B-H2B∙∙∙O2A	2.48	3.211(2)	135	1-x,1/2 + y,3/2-z
**C-X…Cg**	**X…Cg (Å)**	**C-X..Cg (°)**	**Y..Cg (Å)**	**Symmetry code**
C3B-H3B Cg2	2.93	133	3.628(2)	1 + X,3/2-Y,1/2 + Z
**5TPC ACR(7a)**				
O1A-H1A∙∙∙N1B	1.721(19)	2.574(2)	168(2)	

**Table 4:** Hydrogen bond metrics for compounds 8b-14b

**D--–H....A**	**H…A (Ǻ)**	**D…A (Ǻ)**	**D –H…A**	**Symmetry operation**
**5TPC44BIPY (8b)**				
O1 –H1 ∙∙∙N1	1.77	2.6543(16)	179	
**TPC44BIPY(9b)**				
O1 –H1 ∙∙∙N1	1.76(3)	2.629(3)	178(3)	
**5TPC44TMBP(10b)**				
O1 –H1 ∙∙∙N1	1.63(4)	2.576(3)	171(3)	
C9 –H9 ∙∙∙O2	2.44	3.350(3)	166	1-x,-1 + y,1-z
**C-X…Cg**	**X…Cg (Å)**	**C-X..Cg (°)**	**Y..Cg (Å)**	**Symmetry code**
C5-Cl1 Cg2	3.6338(12)	90.93(8)	4.045(3)	X,1 + Y,Z
**TPC44TMBP(11b)**				
O3 –H1 ∙∙∙N1	1.81	2.592(9)	156	
N2 –H2 ∙∙∙O2	1.75	2.569(8)	171	
C20-H20∙∙∙O4	2.59	3.488(10)	163	-1/2 + x,1/2-y,z
**TDC44TMBP(12b)**				
N1 -H1A∙∙∙O2	1.75	2.5883(19)	165	
O3 -H3A∙∙∙O1	1.56(2)	2.5439(16)	176(2)	
C7 –H7 ∙∙∙O4	2.42	3.297(2)	156	1-x,1-y,1-z
C8 –H8 ∙∙∙O4	2.48	3.368(2)	161	1 + x,y,1 + z
C11-H11∙∙∙O1	2.39	3.201(3)	146	-x,1-y,1-z
**5TPC44BIPZ(13b)**				
N1 -H1A∙∙∙O3	1.85	2.7396(16)	167	
N1 -H1B∙∙∙O4	1.99	2.8852(19)	159	2-x,1-y,1-z
O1 -H2A∙∙∙O4	1.52	2.5202(17)	172	
**5TPC44PYNO(14b)**				
O1 -H1A∙∙∙O3	1.84(5)	2.560(4)	171(5)	
C6 –H6 ∙∙∙O2	2.39	3.287(5)	163	
C7 –H7 ∙∙∙O3	2.50	3.381(4)	158	x,1/2-y,-1/2 + z
C10-H10∙∙∙O2	2.51	3.308(4)	144	x,1/2-y,1/2 + z

### Crystal structure description of 5TPCAMPY (1a)

**Figure 3:**
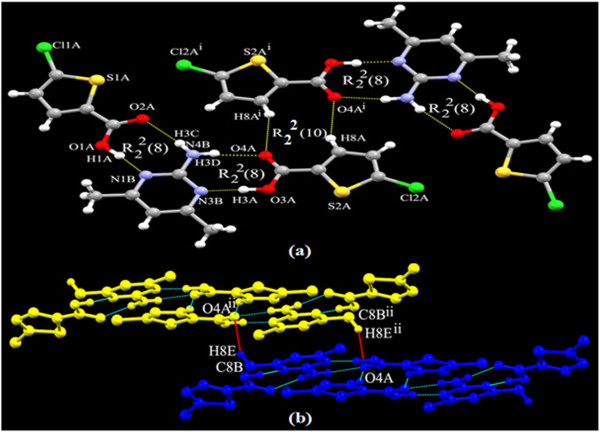
**(a) Formation of hexameric super molecule in (1a) by the N-H…O and C-H…O hydrogen bonds. (b)** Hexameric super molecule of one plane (yellow) linked to the similar kind of hexameric super molecule in another plane (blue) linked by soft C-H…O hydrogen bonds (red dotted lines).

5TPCAMPY crystallizes in P2_1_/c monoclinic space group where the asymmetric unit consists of one molecule of AMPY and two crystallographically independent molecules of 5TPC. The desired primary N-H…O and O–H…N hydrogen bonding interactions between the two carboxylic groups of two 5TPC and the AMPY molecule result in the formation of a heterotrimer (HT) (Scheme 1). The commonly occurring R_2_^2^(8) synthon by hydrogen bonds in the presence of pyrimidine/carboxylic acid interaction is the primary motif. The carboxylic acid moieties are coplanar with respect to the thiophene ring of the 5TPC resulting in a trimer. Adjacent trimers produced from these two strong hydrogen bonds are connected via two symmetry related hydrogen bonds involving H on the thiophene ring and O on the carboxylic acid, (Figure 3a). This results in the formation of hexameric supermolecule with the formation of R_2_^2^(10) synthon (Figure 3a). All these six molecules lie in the same plane they are connected to similar typed six molecules lying on the same plane by a pair of soft C-H…O hydrogen bonds in between the methyl group of pyrimidine and carboxylic acid group of 5-TPC (Figure 3b). There are Cl-π interactions observed between chlorine of thiophene ring and the thiophene ring of the adjacent hexameric supermolecule. These interactions are observed in between Cl1A → Cg3 [symmetry code: -1 + X,1/2-Y,-1/2 + Z] and Cl2A → Cg2 [symmetry code: 1 + X,1/2-Y,1/2 + Z] (where Cg2 = S1A,C2A → C5A; Cg3 = S2A, C7A → C10A).

### Crystal structure description of TPCAMPY (2a)

**Figure 4:**
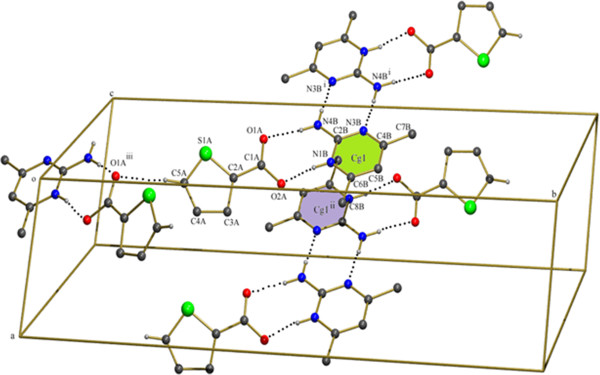
**Formation of R**_**2**_^**2**^**(8) moieties in 2a and the formation of LHT.**

The TPCAMPY crystallizes in the same P2_1_/c monoclinic space group as that of 5TPCAMPY, but the expected neutral molecules were not observed, instead the asymmetric unit consists of a pyrimidinium cation (AMPY^+^) and a thiophene2-carboxylate anion (TPC^-^). In (**1a**) the primary motif is composed of 5TPC and AMPY which is connected through a couple of strong (O–H…N and N-H…O hydrogen bonds), whereas in (**2a**), it is made up of charge-assisted hydrogen bond interactions between the carboxylate and the amino-pyrimidinium moieties through N - H · · · O^-^ and N - H^+^ · · · O^-^ hydrogen bonds. This leads to the formation of R_2_^2^(8) ring motif between them (Figure 4). The secondary synthons involving the N - H · · · N hydrogen bonds are responsible for the formation of the linear heterotetramer (LHT) (Scheme 1). The adjacent heterotetramers next to the carboxylate groups (on both sides) do not lie in the same plane and they are connected by a C-H…O hydrogen bonding interactions between hydrogen of thiophene ring of a LHT and carboxylate oxygen of another LHT (Figure 4). This gives rise to a chain of hydrogen bonds. The pyrimidine ring of a LHT present in a plane, exhibits stacking interactions with another pyrimidine ring lying in parallel planes.

### Crystal structure description of TDCAMPY (3a)

**Figure 5:**
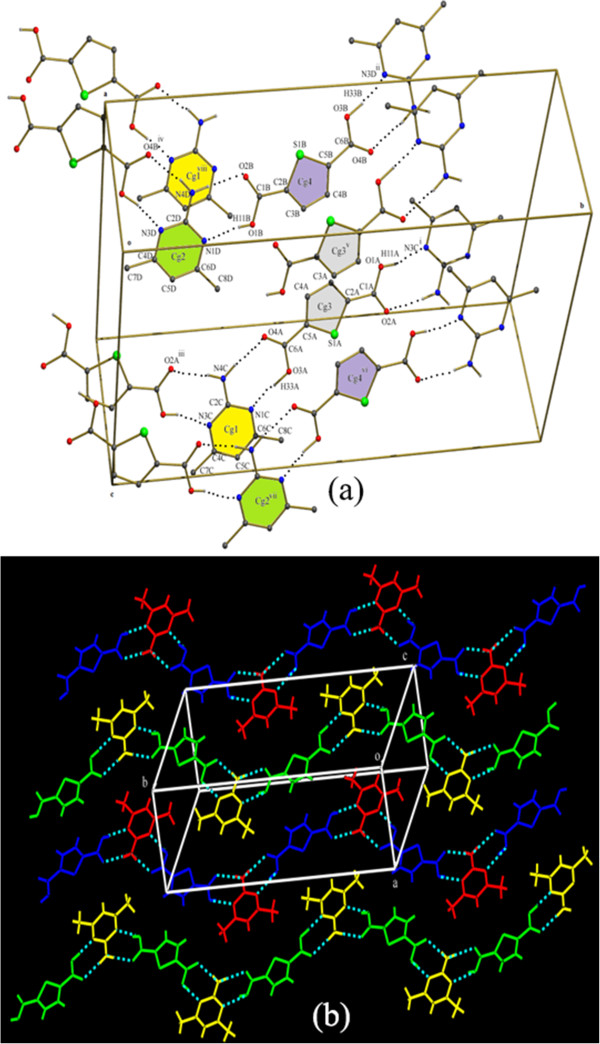
**(a) Formation of supramolecular chains connected by stacking interactions. (b)** Wavy sheet formed through weak C-H···O interaction in TDCAMPY (3a).

As both (**1a**) and (**2a**), **3a** also crystallizes in the same P2_1_/c monoclinic space group. The asymmetric unit consists of two crystallographically independent TDC (A, B) and AMPY (A, B) molecules. The two molecules adopt similar geometry and supramolecular interactions. In the crystal two individual trimers are formed as a result of hydrogen-bond interactions between the two carboxylic acid groups and pyrimidine groups (A molecule of TDC with that of A molecule of AMPY, B molecule of TDC with that of B molecule of AMPY) (Figure 5a). Due to the presence of two carboxylic acid groups of the TDC the chain extends along the b axis. There are stacking interactions observed between the AMPY of A chain and AMPY of B chain, similarly, stacking interactions are observed between TDC of A chain and TDC of B chain (Figure 5a). Two of the AB chains are again connected to an AB chain which are parallel to each other by stacking interaction between the thiophene ring of A chains. This leads to a wavy sheet like arrangement extending along the b axis (Figure 5b).

### Crystal structure description of 5TPCCYT (4a)

The asymmetric unit of (**4a**) consists of a cytosine (CYT), a protonated cytosine (CYTH^+^) and a carboxylate anion. Usually the cytosine gets protonated at N3 and the protonated form (CYTH^+^) can interact with the neutral cytosine (CYT) through a set of three hydrogen bonds generating a (CYTH^+^…CYT) motif as shown in. In the cytosine molecule, protonation at N3 leads to widening of C4-N3-C2 angle from 120.05° to 123.19° [33,34].

**Figure 6:**
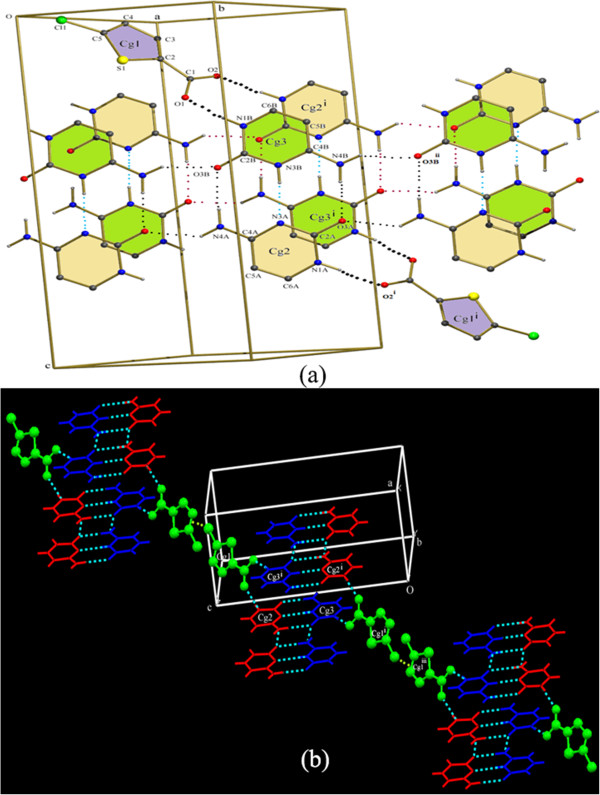
**(a) Base pairing chains in (4a), hydrogens involved in bonding are omitted for clarity. (b)** the base pairing chains linked by Cl…π interactions.

The protonated cytosine (CYTH+), and the cytosine (CYT) are linked via triple hydrogen bonds made up of two N-H…O and a N-H…N hydrogen bond (Figure 6a). This CYT-CYTH^+^ base pair has also been observed in many crystal structures [35,36]. These CYT-CYTH + base pairs are further linked via N-H…O hydrogen bonds leading to chains of base pairs. Such chains running parallel to one another are stacked to the other chain by a pair of π-π stacking interactions between the protonated cytosine (CYTH+) of one chain and the neutral cytosine (CYT) of the other chain (Figure 6a).

Also a pair of strong N-H…O hydrogen bonds between the carboxylate anion and (CYTH + of one chain as well as neutral cytosine (CYT) of the other chain) hold the chains together. This carboxylate anion plays a major role in connecting the chains together. A Cl…π is observed between chlorine of thiophene ring of a chain and the thiophene ring of the adjacent chain (Figure 6b).

### Crystal structure description of 5TPCBA (5a)

**Figure 7:**
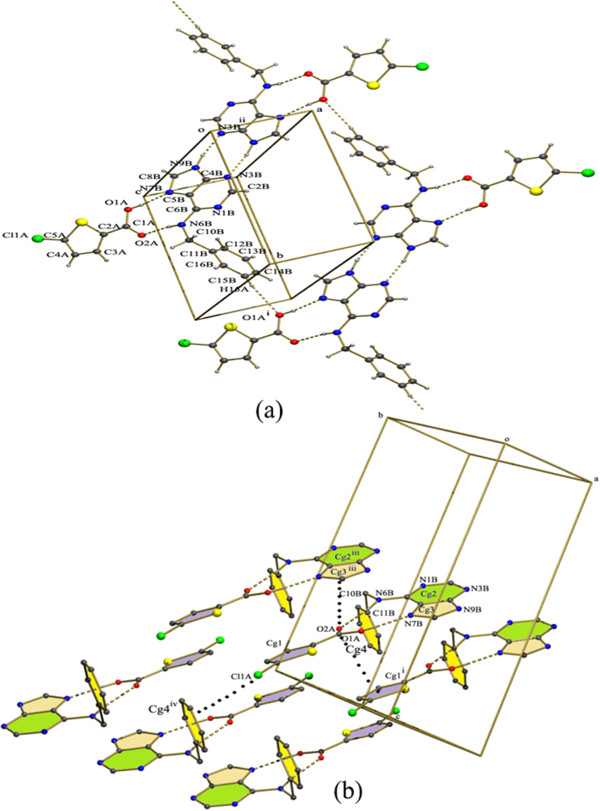
**(a): R**_**2**_^**2**^**(9) motif in (5a) and the formation of tetrameric super molecule. (b)** Adjacent tetrameric super molecules linked by Cl…π interactions between Cl of 5-TPC of one chain and phenyl ring of BA of another chain.

The compound (**5a**) crystallizes in P-1 triclinic spacegroup, where the asymmetric unit consists of one molecule of BA and a molecule of 5TPC. The dihedral angle between the adenine plane and phenyl ring plane is 87.2(3)°. The primary R_2_^2^(9) motif is composed of 5TPC and BA which are connected through a couple of strong O-H…N and N-H…O hydrogen bonds (Figure 7a). This motif is very much similar to that observed in the related structures [37-39]. Adjacent dimeric units are further connected through self complementary secondary N - H · · · N hydrogen bonds. The hydrogen of the phenyl ring and the carboxylic acid oxygen interact in a C-H…O hydrogen bond (Figure 7a). Thus the primary and secondary hydrogen bonds, O - H · · · N, N - H · · · O, N - H · · · N and C-H…O combine to form a tetrameric super molecule (Figure 7a). Each of these tetrameric super molecules is linked by C-H…O interactions. As in the (**4a**), the Cl of the 5-TPC plays a major role in building of the supramolecular architectures. The adjacent chains made of the tetrameric super molecules are linked to one another by the Cl…π interactions between Cl of 5-TPC of one chain and the phenyl ring of BA of another chain (Figure 7b).

### Crystal structure description of 5TPC2NPY (6a)

**Figure 8:**
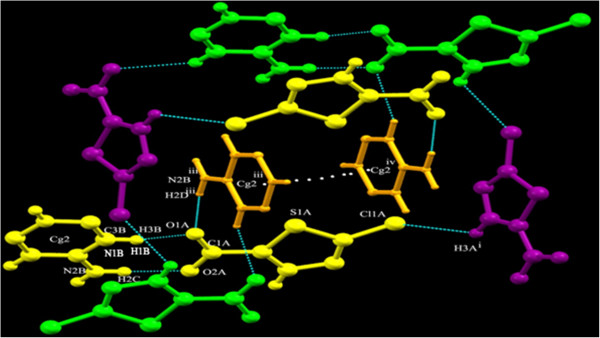
**π-π stacking interaction observed between the 2NPY**^**+**^**pyridinium cations in (6a).**

The compound (**6a**) crystallizes in monoclinic P2_1_/c space group with the asymmetric unit possessing a molecule of 5TPC^-^ carboxylate anion and a molecule of 2NPY^+^ pyridinium cation. The primary motif is very similar to that of (**2a**) and is made up of charge-assisted hydrogen bond interactions between the carboxylate and the amino-pyridinium moieties through N - H · · · O^-^ and N - H^+^ · · · O^-^ hydrogen bonds (Figure 8). These interactions act as the primary hydrogen bonds and are responsible for the R_2_^2^(8) ring motif between them. There is a π-π stacking interaction observed between the 2NPY^+^ pyridinium cations (Figure 8). The C-H…Cl interactions are found between the Cl of a 5-TPC and H of the thiophene ring (Figure 8).

### Crystal structure description of 5TPCACR (7a)

The compound (**7a)** was cocrystallized from methanol and contains a 1:1 mixture of 5-TPC and ACR. O-H · · · N interactions are found between carboxylic acid and the nitrogen containing heterocyclic ring of the acridine. The crystal structure is further stabilized by C—H · · · π interactions. The O—H · · · N hydrogen bond is found between H1A of the carboxylic acid and the N1B nitrogen atom of the acridine (Figure 2a). π-π stacking interactions are found between two oppositely oriented consecutive acridine molecules. Four π-π stacking interactions such as Cg1 → Cg1^v^,Cg1 → Cg2^v^,Cg2 → Cg3^v^ & Cg1 → Cg3^iv^ (Symmetry codes:(iv) -x + 1, -y + 2, -z; (v) -x, -y + 2, -z) stack the acridine molecules (Figure 2a) (Cg1 = centroid of ring N1B/C10B/C11B/C5B/C13B/C12B, Cg2 = centroid of ring C1B/C2B/C3B/C4B/C11B/C10B Cg3 = centroid of ring C6B/C7B/C8B/C9B/C12B/C13B). These π-π stacking interactions of acridine molecules which are also hydrogen bonded to carboxylic acids form a chain along the a axis (Figure 2a). These adjacent chains are cross-linked via intermolecular C—H · · · π interactions involving the S1A/C2A—C5A thiophene ring (centroid Cg4) (Figure 2b).

### Crystal structure description of 5TPC44BIPY and TPC44BIPY (8b, 9b)

**Figure 9:**
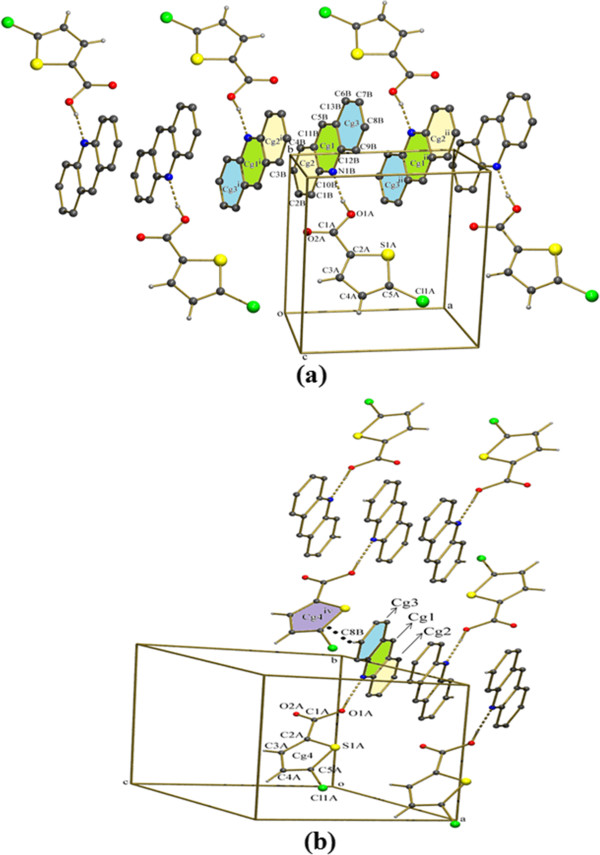
**(a) Stacking interaction between the oppositely oriented consecutive acridine molecules in (7a). (b)** Two of the adjacent chains cross-linked via intermolecular C—H···π interactions.

**Figure 10:**
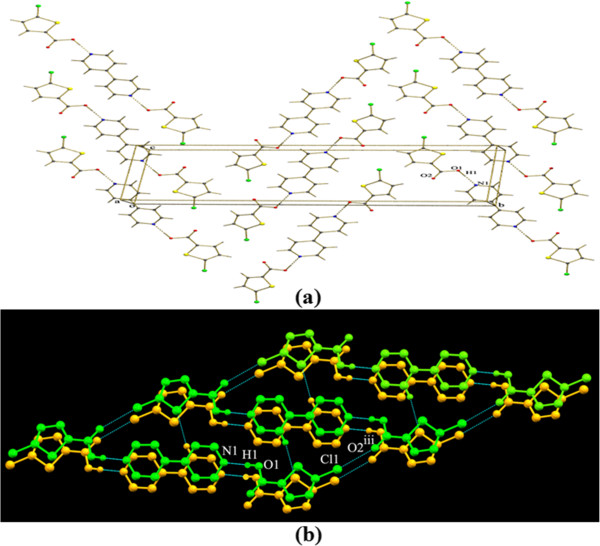
**(a) Formation of ladder through O–H…N and Cl…O bonds in (8b) where 4-4bipy acts as rungs and 5-tpc as uprights. (b)** The three molecule aggregates in 8b are further linked to similar neighboring aggregates through strong Cl…O interactions.

**Figure 11:**
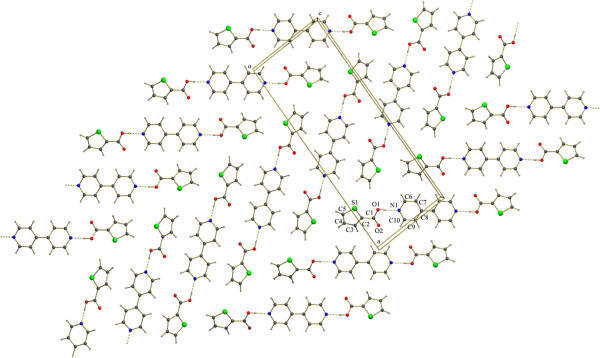
Formation of ladder through C-H…O hydrogen bonds in (9b).

The ORTEP views of 5TPC44BIPY and TPC44BIPY are shown in (Figure 9a and b). The asymmetric unit of (**8b**) is composed of one molecule of 5-TPC and half molecule of 44BIPY. Similarly the asymmetric unit of (**9b**) is composed of one molecule of TPC and half a molecule of 44BIPY. The 44BIPY molecules in both the cases lie about an inversion centre connected by the acid molecules on either side. As expected both the compounds show the three molecules aggregate made up of 44BIPY ligands bridging the two carboxylic acids. Both the compounds reveal supramolecular adducts sustained by symmetric COOH…N_arom_ supramolecular heterosynthon (Scheme 2c) (Figures 10 and 11).

But the expected R_2_^2^(7) synthon (Scheme 2a) that is formed predominantly between pyridine and carboxylic acid groups in similar type compounds is completely absent. This may be due to the least twisting of the carboxyl group attached to the thiophene ring. The carboxyl group is twisted with an angle of 2.6(2)**°** and 4.0(3)**°** in both compounds respectively, with respect to the least squares plane of the thiophene ring. The dihedral angle between the two pyridine rings is 0.0(7)**°**, 35.13(12)**°** (**8b** and **9b**). The three molecule aggregates in (**8b**) are further linked to similar neighboring aggregates through strong Cl…O and soft C-H…O interactions to generate two dimensional arrays in (**8b**) and (**9b**) respectively (Figure 10b). In both the cases the 44BIPY molecules act as rungs between the two chains which serve as uprights of a ladder. In (**8b**) two of these ladders are linked by weak C-H…S interactions, C7-H7....S1 (Figure 10b). Although both (**8b**) and **(9b**) form similar type of ladders, the difference lies in the type of arrangement. In (**8b**) all the three molecules lie in the same plane thereby shaping it into a planar ladder. But in (**9b**) the ladder is bridged by 44BIPY molecules and an adjacent acid which is flanked and slightly away from 44BIPY (Figures 10a and 11). The large deviation of 44BIPY in (**9b**) as well as the presence of Cl of the 5-TPC in (**8b**) are perhaps responsible for the difference in the higher level of supramolecular organization.

### Crystal structure description of 5TPC44TMBP and TPC44TMBP (10b, 11b)

**Figure 12:**
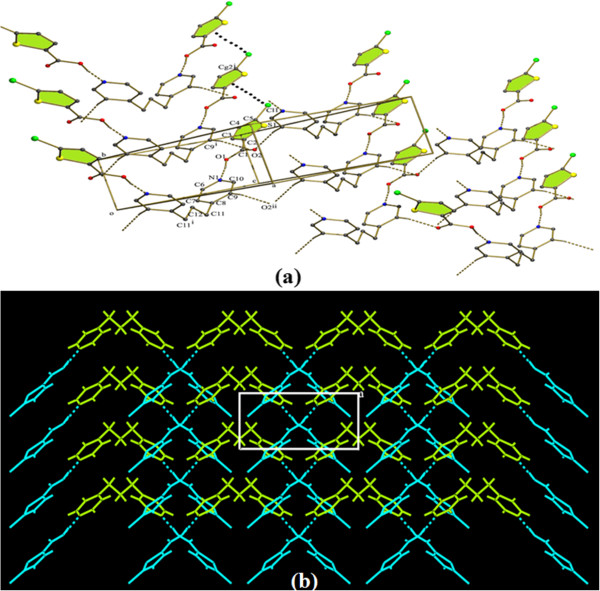
**(a) Formation of ladders by Cl…π interactions. (b)** Supramolecular architectures formed in (10b).

The ORTEP views of (**10b**) and (**11b**) are shown in (Figure 9c and d). The asymmetric unit of (**10b)** is composed of one molecule of 5TPC and half molecule of TMBP. The carboxylic acid-aromatic nitrogen heterosynthon is found on both pyridine rings of the TMBP in (**10b**). The crystal structure of (**11b**) is sustained by carboxylic acid-aromatic nitrogen heterosynthon (Scheme 2c) on both sides of the TMBP. Thus the three molecule aggregate is found in both cases. In (**10b**) the C–O bond distances of the carboxylic acid are 1.212(2) Å and 1.298(3)Å. The short and straight + N2-H2…O2– hydrogen bond could pull that oxygen atom (O2) away from its attached carbon atom (C1) while other C-O bond strengthens and shortens to compensate, and indeed C1-O2 at 1.292(10)Å is the longer bond. Further, the large difference between the exocyclic bond angles (O1-C1-C2 = 123.6(8)Å, O2-C1-C2 = 112.9(7)Å) of the carboxyl group confirms that it is really a carboxyl group and not a carboxylate ion. The dihedral angle between the pyridine ring/carboxylic acid is 7.5(16)°, 2.9(4)° and 7.9(4)° in (**10b**) and **(11b**) respectively. The bipyridine ring is very much bent which can be noted by the very high dihedral angle between the two pyridine rings of the TMBP 78.31(14)° and 51.7(4)° (**10b**) and (**11b**) respectively. As in the case of the (**8b**) and (**9b**) the same type of ladders are formed in (**10b**). In (**10b**) the ladders are formed by Cl…π interactions between the adjacent 5-TPC molecules (Figure 12a).

Two of these parallel ladders are further linked by weak C–H…O interactions on both sides of the TMBP between the carboxylic acid oxygen and the hydrogen of the TMBP, leading to a herring bone like pattern in (**10b**) (Figure 12b).

**Figure 13:**
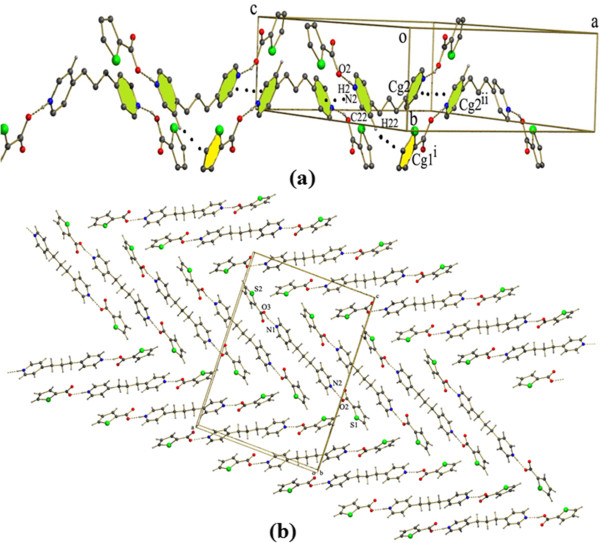
(a) Formation of chain by π-π and C-H…π interactions (b) supramolecular network in (11b).

In **11b** there is a π-π stacking interaction of two oppositely oriented TMBP molecules and this extends into a chain by consecutive similar type π-π stacking interactions (Figure 13). Also there is a C–H…π interaction inbetween the H of the pyridine ring and the oppositely oriented thiophene ring.

### Crystal structure description of TDC44TMBP (12b)

**Figure 14:**
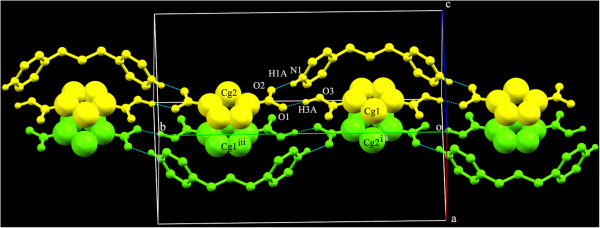
Formation of supramolecular chains (yellow, green) in (12b) by N-H…O and O-H…O interactions and these chains linked by stacking interactions between TDC rings (represented as spacefilled models).

The asymmetric unit of compound (**12b**) consists of one half of trimethylene dipyridinium cation, half a 2,5-thiophenedicarboxylate anion, and half a 2,5-thiophenedicarboxylic acid. The thiophene dicarboxylic acid group possesses two functional groups capable of two-point recognition. But each of the carboxylate groups is involved in two one point supramolecular heterosynthons (Scheme 2c): one with the protonated nitrogen atom of trimethylene dipyridinium (N1-H1A…O2 = 2.5883(19)Å), a second with the adjacent carboxylic acid molecule (O3-H3A…O1 = 2.5439(16)Å). The C-O bond distances are 1.254(2)Å, 1.251(2)Å, and 1.307(2)Å, 1.218(2)Å for the carboxylate and carboxylic acid moiety, respectively. The C-N-C angle of the trimethylene dipyridinium is 121.83(16)°. The combination of these heterosynthons leads to a chain extending along the b axis (Figure 14). Two of these adjacent chains are linked to each other by stacking interactions between the two TDC rings (carboxylate thiophene ring of one chain and carboxylic acid thiophene ring of another) (Figure 14). Similarly two of these chains are linked to two other chains by soft C11-H11…O1 = 3.201(3) (Symmetry code: -x,1-y,1-z).

### Crystal structure description of 5TPC44BIPZ (13b)

**Figure 15:**
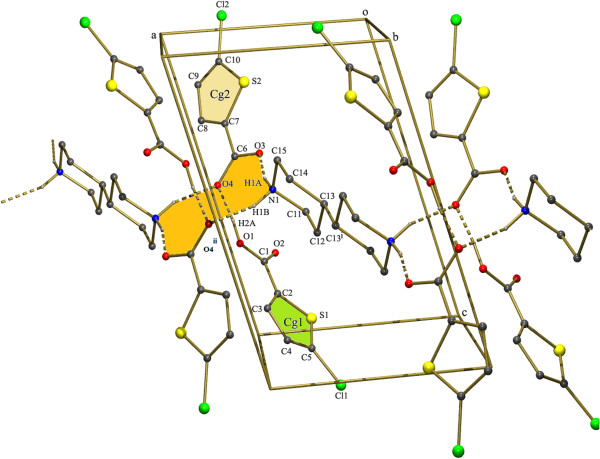
**R**_**4**_^**4**^**(12) ring motif and bridging of the ring motifs by the bipiperazinium cations in (13b).**

The asymmetric unit of compound (**13b**) consists of one half of a bipiperazinium cation, a 5-chloro-thiophene2-carboxylate anion, and a 5-chloro-thiophene2-carboxylic acid. The C-O bond distances are 1.2486(19)Å, 1.2696(19)Å, and 1.314(2)Å, 1.208(2)Å for the carboxylate and carboxylic acid moiety, respectively. The C-N-C angle of the dipiperazinium is 111.33(12°). As observed in (**12b**) the same two one point supramolecular heterosynthons appear (Scheme 2c): one with the protonated nitrogen atom of dipiperazinium (N(1) --H(1A) ..O(3) = 2.7396(16)Å), a second with the adjacent carboxylic acid molecule (O1-H2A…O4 = 2.5202(17)Å). Another N1-H1B…O4 = 2.8852(19) [symmetry code: 2-x,1-y,1-z] is observed between the N of the bipiperazinium cation and the oxygen of the carboxylate. The other end of the bipiperazinium cation containing two hydrogens is also involved in the same kind of interactions. Thus this forms a large R_4_^4^(12) ring motif (Figure 15). These rings motifs extend into a chain linked by the bipiperazinium cation (Figure 15).

**Figure 16:**
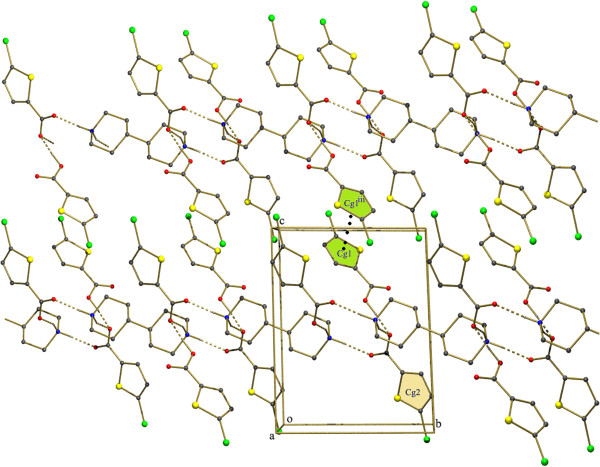
Two of the chains linked by π-π stacking interactions between the 5-TPC rings in (13b).

The carboxylic acid is linked to the carboxylate anion which is perpendicular to it (involved in formation of R_4_^4^(12) ring motif) by a strong O1-H2A…O4 = 2.5202(17) hydrogen bonding interaction. These carboxylic acids thus lie as pendants on both the sides to the chain made up of bipiperazinium cations. Two of these chains are again linked to one another by the stacking interactions between two carboxylic acids of each chain (Cg1-Cg1^iii^ where Cg1 = S1,C2,C3,C4,C5 [symmetry code iii = 2-x,1-y,1-z]) (Figure 16).

### Crystal structure description of 5TPC44PYNO (14b)

**Figure 17:**
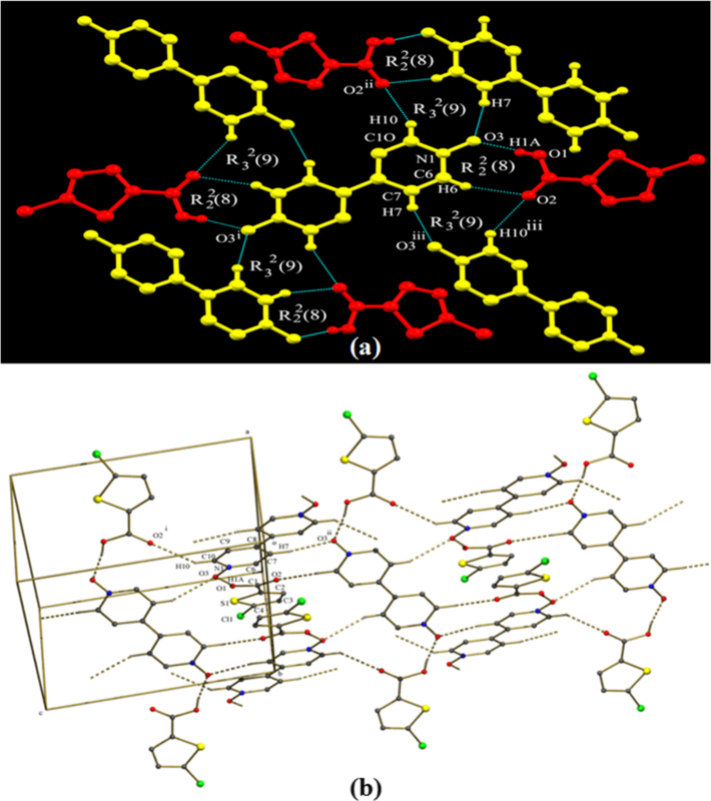
**(a) A portion of the crystal packing of 14b showing hydrogen-bonding patterns with graph-set notations R**_**3**_^**2**^**(9), R**_**2**_^**2**^**(8).** Dotted lines denote hydrogen bonds. H atoms non-involved in hydrogen-bonding omitted for clarity. **(b)** Hydrogen bonded network formed in (14b).

Similar to the preceding 44BIPY and TMBP compounds (**8b, 9b,** and **10b)**, compound (**14b**) crystallizes with a 1:2 ratio of acid and bipyridine components. The acid group and the PYNO ring lie on the same plane, the dihedral angle between them being 4.48(16)°. One half of the PYNO ring connects with four other molecules (two 5-TPC molecules and two PYNO molecules) by a series of C-H…O and O–H…O hydrogen bonds. Thus this gives rise to two sets of R_2_^2^(8) and R_3_^2^(9) on each side of the PYNO ring (Figure 17a). Each of the N^+^-O^-^ functions plays a significant role by participating in bifurcated interactions to neighboring molecules; hence there is a formation of network. The network is further stabilized by a π-π stacking interaction between the thiophene ring and the PYNO ring (Cg1-Cg2^iv^ where Cg1 = N1,C6,C7,C8,C9,C10 and Cg2 = S1,C2,C3, C4, C5 [Symmetry code (iv) = -X,1-Y,-Z] (Figure 17b).

As said earlier, carboxyl and pyridyl functional groups are known to form the most robust intermolecular interactions. The occurrence of synthons III and IV are accompanied by a strong N-H…O or O–H…N and weaker C-H…O hydrogen bonds (Scheme 2**a, b**). This leads to the formation of R_2_^2^(7) synthon. Previous reports [40] say that for the formation of this motif, strong geometrical complementarity between base and acid molecules is necessary. Our reports add value to this point, since there is a formation of a noncyclic motif of type V and VI in (**4a, 5a, 7a-9a, 8b-13b**) (Scheme 2c, d). In compounds (**8b, 9b, 12b** and **13b**) the dihedral angle between the carboxylic acid group and the pyridyl group is relatively large range (Table 5), which may probably be the reason for the formation of non cyclic single point synthon. Also the conformational flexibility of the molecules (dihedral angles of two pyridine rings) affects the formation of this synthon which is the case in compounds (**9b-12b**). The value is less for 14b which also explains the reason for non formation of synthon of type V or VI (Scheme 2c, d).

**Table 5:** Comparisons of dihedral angles (between carboxylic acid and pyridine) and C-N-C bond angles (involved in acid–base interaction) in compounds 1a-14b

**Compound**	**Acid**	**Base**	**Synthon**	**Dihedral angle between carboxylic acid and pyridine**	**Dihedral angle between two pyridine rings**	**Acid–base interaction involved C-N-C bond angle**
**1.**	5TPC	AMPY	HT	14.2(15), 9.4(5)		116.89
						117.69
**2.**	TPC	AMPY	LHT	19.1(4)		118.80
						117.30
**3.**	TDC	AMPY	HT	1.8(13), 2.4(12)		117.76
						117.76
						117.92
						118.45
**4.**	5TPC	2NPY	HD	8.5(3)		122.01
						123.94
						121.43
						119.49
**5.**	5TPC	CYT	HD	57.0(2)		103.18
**6.**	5TPC	BA	HD	13(3)		117.84
**7.**	5TPC	ACR	HD	63.6(8)		120.06
**8.**	5TPC	44BIPY	HD	29.68(10)	0.00(7)	117.23
**9.**	TPC	44BIPY	HD	15.6(3)	35.13(12)	117.58
**10.**	5TPC	44TMBP	HD	7.5(16)	78.31(14)	116.38
**11.**	TPC	44TMBP	HT	2.9(4), 7.9(4)	51.7(4)	117.25
						117.85
**12.**	TDC	44TMBP	HD	69.16(1)	82.15	121.82
**13.**	5TPC	44BIPZ	HD	59.06(3)	0.00(1)	111.32
**14.**	5TPC	44PYNO	HD	5.34(3)	0.00(1)	119.93

**Figure 18:**
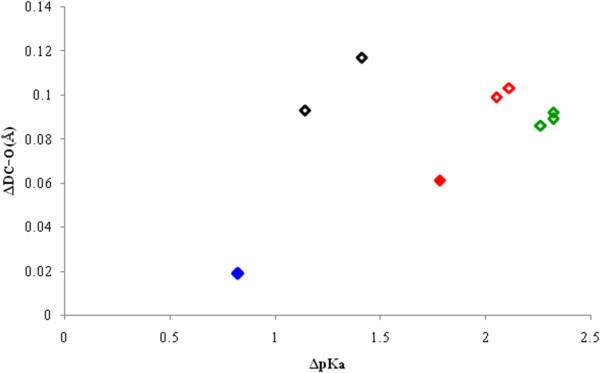
**Plot of ΔpKa vs ΔDc-o for compounds.** Blue (cytosine salt) red block (ampy salt) red box (ampy cocrystal) black (bipy cocrystal) green (44TMBP cocrystal).

Among the 14 structures reported here, 8 of them are co-crystals while the remainder are salts. Several papers have also reported and used the acid dissociation constant pK_a_ in predicting the formation of salts/cocrystals [41]. In our recent report involving pyrimidine with various other acids we have tried to rationalize the formation of salt/cocrystals in terms of pK_a_ values. Also in our previous report we presented a plot of ΔpKa vs ΔDc-o, where the X-axis corresponding to the ΔpKa and Y-axis corresponding to ΔD_C-O_ [where ΔpKa = pKa(base) - pKa(acid), ΔD_C-O_ = difference between the lengths of the two C - O bonds in a carboxyl group]. Now we present a same kind of plot using the calculated ΔpKa [42] of the molecular compounds (**1a-4a, 8b-12b**) and measured ΔD_C-O_ thiophene carboxylic acids used here (Figure 18). The C - N - C bond angle in AMPY and CYT, which involves acid–base interaction, ranges from 116 to 118° in most cases (indicative of co-crystal formation) and increases to a higher range of 119 - 124° indicating the formation of salts (Table 5). The scattergram indicates the densely populated boxes on the upper side which corresponds to co-crystals having larger ΔD_C-O_ and the scarcely populated blocks on the bottom side which corresponds to formation of salts. Also from the plot it can be seen that each of the populated areas corresponds to each type of base involved in salt/cocrystal formation.

## Experimental

### Materials and methods

Commercial starting materials were used without further purification. 2-amino-4,6-dimethylpyrimidine, TPC, TDC, ACR,TMBP, 44BIPY, were purchased from Sigma-Aldrich, 5-Chloro thiophene 2- carboxylic acid (Hoechst Aktiengesellschaft), methanol/ethanol (Qualigens, India) were used (Scheme 3). IR spectra of the compounds in region 400–4000 cm^-1^ were recorded as pressed disks (1% by weight in KBr) on a Shimadzu FT IR spectrophotometer.

### Preparation of compounds (1a-7a,8b-14b)

Compounds (**1a-3a**) were prepared by mixing hot methanolic solution of AMPY with hot methanolic solution of 5-TPC/TPC/TDC in 1:1 molar ratio and were allowed to warm over a water bath for half an hour. The mixtures were cooled slowly and kept at room temperature. After a few days, colorless prismatic crystals of (**1a-3a**) separated out of the mother liquor. Compounds (**4a - 7a**) were prepared by mixing hot methanolic solution of 5TPC with hot methanolic solution of CYT/BA/2NPY/ACR in 1:1 molar ratio and were allowed to warm over a water bath for half an hour. The mixtures were cooled slowly and kept at room temperature. After a few days, colorless prismatic crystals of (**4a,5a**), yellow prismatic crystals of (**6a**) and yellow plate like crystals of (**7a**) separated out of the mother liquor. IR selected bands for (**1a**) (cm^-1^): 3346(s), 3174(m), 2924(m), 2852(m), 2378(m), 1660(s), 1589(s), 1531(s), 1421(s), 1330(s), 1274(s), 1101(s), 1060(s), 993(s), 815(s), 756(s), 574(s), 518(s), 466(s). IR selected bands for (**2a**) (cm^-1^): 3334(m), 3169(m), 3074(m), 1672(s), 1604(s), 1517(s), 1417(s), 1375(s), 1321(s), 1107(s), 1029(s), 964(s), 860(s), 823(s), 771(s), 738(s), 597(m). IR selected bands for (**3a**) (cm^-1^): 3344(s), 2924(m), 2374(s), 1658(s), 1597(s), 1527(s), 1438(s), 1350(s), 1276(s), 1024(s), 833(s), 756(s), 667(s), 576(s). IR selected bands for (**4a**) (cm^-1^): 3375(m), 3101(m), 1683(s), 1658(s), 1531(s), 1433(s), 1332(s), 1278(s), 1236(s), 1105(s), 1004(s), 812(s), 752(s), 669(s), 493(s). IR selected bands for (**5a**) (cm^-1^): 3282(s), 1627(s), 1415(s), 1330(s), 1290(s), 1138(s),1103(s), 997(s), 752(s), 638(s), 526(s). IR selected bands for (**6a**) (cm^-1^): 3259(s), 3003(m), 2526(m), 1674(s), 1531(s), 1487(s), 1431(s), 1365(s), 1253(s), 1157(s), 1051(s), 977(s), 792(s), 761(s), 621(s), 514(s). IR selected bands for (**7a**) (cm^-1^): 3402(m), 1685(s), 1427(s), 1327(s), 923(s),736(s), 601(s), 445(s).

Similarly compounds (**8b,9b**) were prepared by mixing hot ethanolic solution of 44BIPY with hot ethanolic solution of 5TPC/TPC in 1:1 molar ratio and were allowed to warm over a water bath for half an hour. The mixtures were cooled slowly and kept at room temperature. After a few days, colorless needle typed crystals of (**8b,9b**) separated out of the mother liquor. IR selected bands for (**8b**) (cm^-1^): 3425(m), 2924(s), 2374(s), 1654(s), 1595(s), 1421(s), 1325(s), 1265(s), 999(s), 806(s), 744(s), 624(s), 462(s). IR selected bands for (**9b**) (cm^-1^):3107(m), 1691(s), 1600(s), 1525(m), 1406(s), 1357(s), 1274(s), 1056(s), 1006(s), 813(s), 756(s), 717(s), 626(s), 459(s).

Compounds (**10b-12b**) were prepared in same procedure where hot ethanolic solution of TMBP was mixed with hot ethanolic solution of 5TPC/TPC/TDC in 1:1 molar ratio. After a few days, colorless plate type crystals of (**10b-12ba**) separated out of the mother liquor. Similarly (**13b, 14b**) were prepared by mixing hot ethanolic solution of 5-TPC with hot ethanolic solution of 44BIPZ/44PYNO in 1:1 molar ratio and were allowed to warm over a water bath for half an hour. The mixtures were cooled slowly and kept at room temperature. After a few days, colorless needle type crystals of (**13b**) and pale yellow prismatic crystals of (**14b**) separated out of the mother liquor. IR selected bands for (**10b**) (cm^-1^): 3427(m), 1612(s), 1429(s), 1026(s), 810(s), 987(s), 810(s), 758(s), 518(s), 460(s). IR selected bands for (**11b**) (cm^-1^):3448(m), 1681(s), 1614(s), 1523(s), 1415(s), 1357(s), 1286(s), 1215(s), 1031(s), 821(s), 759(s), 640(s), 501(s), 455(s). IR selected bands for (**12b**) (cm^-1^): 3452(m), 1683(s), 1658(s), 1521(s), 1419(s), 1207(s), 1105(s), 1056(s), 815(s), 750(s), 580(s), 509(s). IR selected bands for (**13b**) (cm^-1^): 2960(s), 2490(s), 1687(s), 1589(s), 1533(s), 1436(s), 1332(s), 1278(s), 1107(s), 1006(s), 921(s), 813(s), 748(s), 669(s), 528(s), 491(s). IR selected bands for (**14b**) (cm^-1^): 3423(m), 3076(s), 2405(s), 1687(s), 1535(s), 1477(s), 1411(s), 1330(s), 1271(s), 1213(s), 1184(s), 1026(s), 995(s), 947(s), 810(s), 748(s), 551(s), 466(s).

### Crystal structure determination

Intensity data sets were collected at room temperature, on a BRUKER SMART APEXII CCD [43] area-detector diffractometer equipped with graphite monochromated Mo Kα radiation (λ = 0.71073 Å). The data were reduced by using the program SAINT [43] and empirical absorption corrections were done by using the SADABS [43]. The structures were solved by direct methods using SHELXS-97 [44] and subsequent Fourier analyses, refined anisotropically by full-matrix least-squares method using SHELXL-97 [44] within the WINGX suite of software, based on F^2^ with all reflections. All carbon hydrogens were positioned geometrically and refined by a riding model with U_iso_ 1.2 (1.5 for methyl groups) times that of attached atoms. All non H atoms were refined anisotropically. The molecular structures were drawn using the ORTEP-III [45], POV-ray [46] and MERCURY [47]. The crystals remained stable throughout the data collection. The dihedral angles were determined using PLATON using the experimental results reported here. The differences in theta values with that of expected values for compounds 8b and 11b are due to the poor quality of crystals.

## Conclusions

Structural studies of these 14 supramolecular compounds of N-heterocyclic bases and various thiophene carboxylic acids show differences depending on the number of proton acceptors (nitrogen atoms) in the base molecule. Of the 14 compounds carboxylic acid to pyrimidine/pyridine proton transfer has occurred in five of these compounds. The (HT) and (LHT) are dominant in the crystal structures of the adducts containing N-heterocyclic bases with two proton acceptors (**1a-7a**) in the heterocyclic ring and thiophene carboxylic acids. N-heterocyclic bases with one proton acceptor in the heteroyclic ring (**8b-14b**) form a linear one point hetero synthon (HD) with thiophene carboxylic acids. Also in presence of the classical complementary pair of O - H · · · N/O^-^ · · · H - N^+^ and N - H · · · O^-^/N - H · · · O hydrogen bonds the non classical interactions like Cl…π, C-H…Cl, C-O…Cl, C-H…S, Cl…Cl and C-H…π take part in determining the supramolecular architectures. The majority of the compounds showed π-π stacking between the pyrimidine-pyrimidine, pyridine-pyridine and acid-acid moieties rather than acid-pyridine or acid-pyrimidine moieties. The compounds involving bipyridine and bipyridine type ligands mostly show the three molecules aggregate made up of bipyridine/bipyridine type ligands bridging the two carboxylic acids. The above results and the synthetic strategies can also be employed practically to other biologically relevant systems.

### Supplementary material

CCDC 973226–973239 contain the supplementary crystallographic data for the complexes [**1a-7a**], [**8b-14b**] respectively and can be obtained free of charge via http://www.ccdc.cam.ac.uk/conts/retrieving.html, or from the Cambridge Crystallographic Data Center, 12 Union Road, Cambridge CB2 IEZ, UK; fax:(+44)1223-336-033; or e-mail: deposit@ccdc.cam.ac.uk.

## Competing interests

The authors declare that they have no competing interests.

## Authors’ contributions

This work was prepared in the research group of PTM. He proposed the work and drafted the manuscript. SJJ participated in the design and presided over the experiments, collected the X-ray data and drafted the manuscript. Both authors read and approved the final manuscript.
